# Observations on the Climacteric Disease, with Cases

**Published:** 1844-12

**Authors:** Henry Kennedy

**Affiliations:** One of the Medical Officers of St. Thomas’s Dispensary


*Observations on the Climacteric Disease, with Cases.* By Henry Kennedy, M.B. &c. &c. One of the Medical Officers of St. Thomas's Dispensary.—It is now more than twenty years since Sir Henry Halford published his paper on the climacteric disease, and for many a long year we have directed our attention to the malady in question, but not with the most satisfactory results. We occasionally see people, at all ages, droop in general health, but without any tangible or appreciable disease of any particular organ—exhibiting a declension of all the powers of life—a deterioration of all the vital functions—and this state continuing for many months, ending in death, or a gradual restoration to health. Generally, however, some one organ, as the heart, the lungs, the kidneys, or the stomach gives way before the fatal termination arrives, and appears to be the immediate cause of death. That such a break-up of the constitution takes place more frequently at 63, than at 53—43, or 33, might be naturally expected; but beyond this, we verily believe that there is nothing about the "Grand Climacteric" except the force of imagination. Dr. Kennedy believes that the malady in question is a substantive disease; but that it is not confined to the climacteric period, as he has seen it not unfrequently between the age of twenty and thirty years. But this is not all. Our author observes:—*Were I to speak from my own experience, I should say that the persons who pass through life without having labored under it, once, if not twice, are the exceptions to the general rule.*" The following sketch will show that the climacteric disease is just what is familiarly called being "out of health," or "out of condition," for a longer or shorter period.

"Climacteric disease in general commences in a very gradual way. From three to six weeks may pass over, the individual not feeling quite well, and yet not making any distinct complaint. I have known it happen too, though it is not common, that the patient was observed by his friends to be looking ill for a considerable time before he made any complaints whatever. In the great majority of cases, however, the remarkable change the countenance undergoes is not observed till a later period of the disorder. Pains of one form or other are among the most common symptoms ushering in the attack; these may be of a darting and transient character, passing through the entire frame, or they may be more fixed and confined to a certain part. In the former case they are set down as rheumatic or gouty pains, according to the habits, or, it may be, the wishes of the individual; while in the latter nothing remarkable is to be observed about them, except that in general they are in a very marked degree periodic. Another very common symptom complained of in the earlier stages of the disorder is weakness, which is referred in general to the knees, the patient expressing himself in the usual way, by saying that these parts feel as week as water. It is not alone when the patient is walking about that this weakness is complained of; on the contrary, they suffer from it while lying on a sofa, and I have seen it complained of to such a degree that it was described as amounting to absolute pain. It is also worthy of remark, in connexion with this sense of weakness, that it does not seem to be increased by any exercise the individual may take. In one instance, which will be given in detail, this symptom recurred for several days, and at a particular time of each day, before any other symptom showed itself, the individual during this period wondering what could be the cause of such weakness.

"It has been already stated that the disease under notice generally commences in a gradual way. To this, however, there are some remarkable exceptions, and this is an important point to keep in mind. I have known the disease commence with what may be called acute symptoms. Thus a common bilious attack has been followed at once by the usual symptoms, and well-marked climacteric disease has been established within fourteen days. A case of this sort will be detailed. A common cold, or influenza, has been already mentioned as ushering in the attack. But probably the most important of this class of cases is where the disease commences with head symptoms of so acute a character as to throw the medical man entirely off his guard. Under such circumstances a wrong view is very apt to be taken of the case, and erroneous treatment accepted in consequence. This will be alluded to again.

"After the pains, which have been before spoken of, have existed some time, other symptoms make their appearance, in quicker succession too than the commencement of the disease would lead one to expect. The appetite begins to fail; this soon increases to a total loss of it; and finally, should the attack be a well marked one, an utter aversion for all sorts of food succeeds. With this there is of course loss of flesh, and strength, both of mind and body, and above all, the sleep at this period goes astray. In a disease which is marked by such a variety of symptoms there is no more constant one than this loss of sleep; one exception only has come under my notice where it did not exist."

Thus the three chief symptoms are loss of flesh, alteration of countenance, and quickened circulation, with disordered function in one or more organs of the body. Or, to use the words of our author, the climacteric disease is—"a decay, for the time, of the several functions constituting life, but more particularly of those of the nervous system. To any one studying a case of the disease, it appears as if the system had got tired, and could not carry on its various functions with its wonted energy." If this be the climacteric disease, we agree with Dr. Kennedy, that it is to be met with at all periods of life, and in all classes of society—but more frebuently, of course, in the later than in the earlier periods of existence.

In respect to treatment, it is hardly necessary to say that no specific plan can be laid down for a disease that has no specific character or seat. Debility is, no doubt, a prominent feature in the malady; but there is so often a local affection attending the general weakness, that we ought always to examine the head, chest, and abdomen. Such a state of bad health as is described by Dr. K. presents usually a considerable degree of derangement of the abdominal secretions; and if these are not corrected, tonics or stimulants will be of little avail. Alteratives, tonics, and change of air, are among the chief therapeutic agents.—*Dublin Journal.*

